# The effect of quality of segmental bowel preparation on adenoma detection rate

**DOI:** 10.1186/s12876-019-1019-8

**Published:** 2019-07-08

**Authors:** Rui Guo, Yong-Jun Wang, Mo Liu, Jun Ge, Ling-Ye Zhang, Ling Ma, Wen-Yu Huang, Hui-Hong Zhai

**Affiliations:** 1 Department of Gastroenterology, Beijing Friendship Hospital, Capital Medical University, 95 Yongan Street, Xicheng Area, Beijing, 100050 People’s Republic of China; 2 Beijing Key Laboratory for Precancerous Lesion of Digestive Diseases, Beijing, 100050 China; 3 National Clinical Research Center for Digestive Diseases, Beijing, 100050 China; 4 Department of Gastroenterology, Beijing Shijingshan Hospital, Teaching Hospital of Capital Medical University, Beijing, 100043 China; 5 National Clinical Research Center of Digestive Diseases, Beijing Friendship Hospital, Capital Medical University, Beijing, 100050 China

**Keywords:** Bowel preparation, Bowel bubble scale (BBS), Boston bowel preparation scale (BBPS), Adenoma detection rate (ADR), Advanced adenoma detection rate (AADR)

## Abstract

**Background:**

The effectiveness in surveillance colonoscopy largely depends on the quality of bowel preparation. We aimed to investigate the quality of bowel preparation segmentally and its effect on Adenoma Detection Rate (ADR) and Advanced Adenoma Detection Rate (AADR) at corresponding bowel segments.

**Methods:**

This is a single-centered and cross-sectional study. A consecutive of 5798 patients who underwent colonoscopy examination were included. Bowel preparation was evaluated based on Bowel Bubble Scale (BBS) in general and Boston Bowel Preparation Scale (BBPS) in each segment (right side, transverse and left side of colon) and total BBPS scores. The quality of bowel preparation was correlated with ADR and AADR.

**Results:**

Four thousand nine hundred forty colonoscopies (14,820 bowel segments) were included in the final analysis. In which 30.9% scored 3, 57.5% scored 2, 11.2% scored 1 and 0.4% scored 0 on basis of BBPS. For each score, ADR were 10.8, 7.7, 4.9 and 3.2%, respectively; whereas AADR were 4.5, 2.8,1.8 and 1.6% (*P* < 0.05). 36.9% of the colonoscopies showed presence of minimal bubbles and 34.3% with no bubble. For bowels without bubbles and with a large amount of bubbles, ADR were 28.3 and 20.0% respectively; and AADR were 13.3 and 7.1% respectively.

**Conclusions:**

Segmental bowels’ cleanliness and the amount of bubbles in bowels significantly affect ADR and AADR. The better the bowel preparation at each segment is and the less bubbles in the bowel there are, the higher ADR and AADR we got. We suggest repeating colonoscopy if any segment of the bowel preparation is poor, or if there is more bubbles, even if the total score of BBPS indicates good or fair bowel preparation.

## Background

Colorectal cancer is the 3rd most common cancer and the 4th leading cause of cancer-related mortality globally [1]. According to the National Cancer Report of China in 2015, it is the 5th most common cancer in morbidity and mortality in China [2], and its incidence is rising each year. Surveillance colonoscopy is an important method of colorectal cancer screening. The effectiveness of adenoma detection in surveillance colonoscopy largely depends on the quality of colonoscopy [3–7]. There are many factors contributing to good quality of colonoscopy, one of which is the quality of bowel preparation. Many studies proved the correlation of good bowel preparation to high adenoma detection rate (ADR) [8, 9]. American College of Gastroenterology (ACG), American Gastroenterological Association (AGA), and American Society Gastrointestinal Endoscopy (ASGE) had jointly published a guideline on bowel preparation before colonoscopy to assist endoscopists in assessing the quality of bowel preparation objectively, and provide recommendations of various methods to optimize bowel preparation based on available clinical evidences [10–12].

Previous studies have focused almost exclusively overall cleanliness of the whole colon and rectum. Contrary to that, there are few studies focusing on the association of individual segment of bowel preparation with ADR and Advanced Adenoma Detection Rate (AADR). Our aim is to establish the evidence in correlating the quality of bowel preparation of the individual bowel segment to the ADR and AADR of the same bowel segment.

The presence of luminal bubble in colonoscopy may affect the ADR and AADR [13]. The routine use of simethicone during endoscopy procedure is not well established in China due to the lack of awareness of such practice. Assessing the effect of luminal bubble to ADR and AADR in the study may assist us in providing the evidence to recommend and justify the routine use of simethicone during the procedures in the country [14, 15].

## Methods

### Study population

5798 patients who underwent colonoscopy were included in the study. The exclusion criteria are: 1.Patients did not receive oral bowel cleaning agent (including cleansing enema or fasting). 2.Patients whose age was under 18 years old, patients with active psychiatric illness, patients who were incompetent in giving consent, multiple comorbidities with American Society of Anaesthesiology (ASA) class 3 or more, patients on anticoagulation which preclude biopsyprocedures, incomplete demographic data, withdrawal colonoscopy time < 6 min, previous colectomy. 3.Failed completion of colonoscopy including poor bowel preparation (not able to identify the direction of the intestine), technical difficulty (physiologic morphological abnormality of colon, effect of intestinal peristalsis, patients with obesity), patients intolerant to colonoscopy procedure, pathological stricture or external compression leading to failure in completion of colonoscopy. This study was approved by Ethic Committee of Beijing Friendship Hospital. (Approval No. 2016-P2–107-01).

### Study design

This is a single-centered and cross-sectional study. For each colonoscopy, the examination was done up to the caecum. Detailed examination of the colonic lumen was done during withdrawal, with assistance of flushing and suctioning aiming to cleanse the bowel. All examinations were performed using the CF-H260AI or CF-H260AL colonoscope, OLYMPUS CV26 processor and CLV260 light source.

All participating endoscopists fulfilled the requirements and credentials of standard guideline [16, 17]. The endoscopists were permitted to use Narrow Band Imaging (NBI) or chromoendoscopy examination at their discretion to perform detailed characterization of polyps. Polyps were then examined by an appointed pathologist.

All participating endoscopists were trained including 8 min of Boston Bowel Preparation Scale (BBPS) educational video (http://domweb.bumc.bu.edu/bowelprep/), and have completed online assessment at the same website. These measures were undertaken to optimize the reliability, reproducibility and accuracy of the colonoscopic evaluation in the center.

Bowel preparations for all patients were done using standard split dose regimen of PEG (Shenzhen Wanhe Pharmaceutical Co. Ltd., Shenzhen, China). Patients were instructed to take low residue diet 1 day before colonoscopy. Then they received a total of 3 L of PEG. They were instructed to drink half of the volume the night before colonoscopy, and the other half 4–6 h before the procedure by a dose of 250mls every 10 min [18].

### Data collection

Demographic and procedural data for each patient were collected including sex, age, time and date of colonoscopy, indication of colonoscopy, withdrawal time of colonoscopy, seniority of operator classified as senior, intermediate, and junior based on numbers of colonoscopy performed in the past (“senior” with lifetime colonoscopy experience of > 10,000, “intermediate” of 5000–10,000, “junior” of 1000–5000). Withdrawal time was defined as the time taken for withdrawal from caecum to anus while examining the colonic mucosa, inclusive of time used for washing and aspirating the bowel residues for colonic examination; the time taken for biopsy and polypectomy were not included in the withdrawal time.

The cleanliness scoring of each bowel segment examined (right side, transverse and left side of colon) was graded by BBPS. The BBPS involves assigning each of 3 regions of the above mentioned a score from 0 to 3 (0 as non-prepared and 3 as perfectly clean). Each segment score is summed for a total BBPS score ranging from 0 to 9 (score 9 as a perfectly clean colon and 0 as a non-prepared colon) [19]. At the same time, the amount of luminal bubbles was assessed based on Bowel Bubble Scale (BBS) (a scoring derived from capsule endoscopy examination) [20]. The amount of luminal bubbles was measured using a 4 points scale in which 0,1,2,3 represent no air bubble, minimal bubbles (≤25% obscured view), moderate amount of air bubbles (25–50% obscured view) and large amount of air bubbles (≥50% obscured view) respectively.

A detailed documentation for detected polyp(s) was done. The number, site, size (visual comparison with forceps of a known dimension and/or visualization after retrieval), morphology (pedunculated, sub-pedunculated, sessile or flat), method of polypectomy (forceps, snaring, endoscopic mucosal resection or endoscopic submucosal dissection), and pathological type of the polyp using narrow band imaging (NBI) or Chromoendoscopy technique, were all recorded.

Polyp(s) found during colonoscopy, along with the character of the lesion(s) were further classified into high risk and low risk as per international consensus: Low risk polyp was defined as 1–2 adenomatous polyp with size < 10 mm. High risk polyp was defined as ≥3 adenomatous polyps, or any polyp size with > 10 mm, or any polyp with more than 1/3 volume of tubulovillous or high grade dysplasia [21].

### Statistical analysis

Data were analyzed using SPSS19.0. Ages were described as mean ± standard deviation (SD). ADR and AADR were described in percentage. Using a generalized estimating equations model, ADR and AADR of each group was adjusted based on sex, age, indication of colonoscopy, time of procedure, and seniority of endoscopists. Multiple comparisons were made between BBPS segment scores to detect the difference in ADR and AADR.95% confidence interval and the *P* value were evaluated. We use Chi-square to calculate the difference in ADR and AADR and ANOVA method to calculate the difference in BBPS and BBS, taking into consideration of seniority of endoscopists. *P*-value of < 0.05 was considered as a statistically significant value.

## Results

### Patient and colonoscopy characteristics

**Fig. 1:**
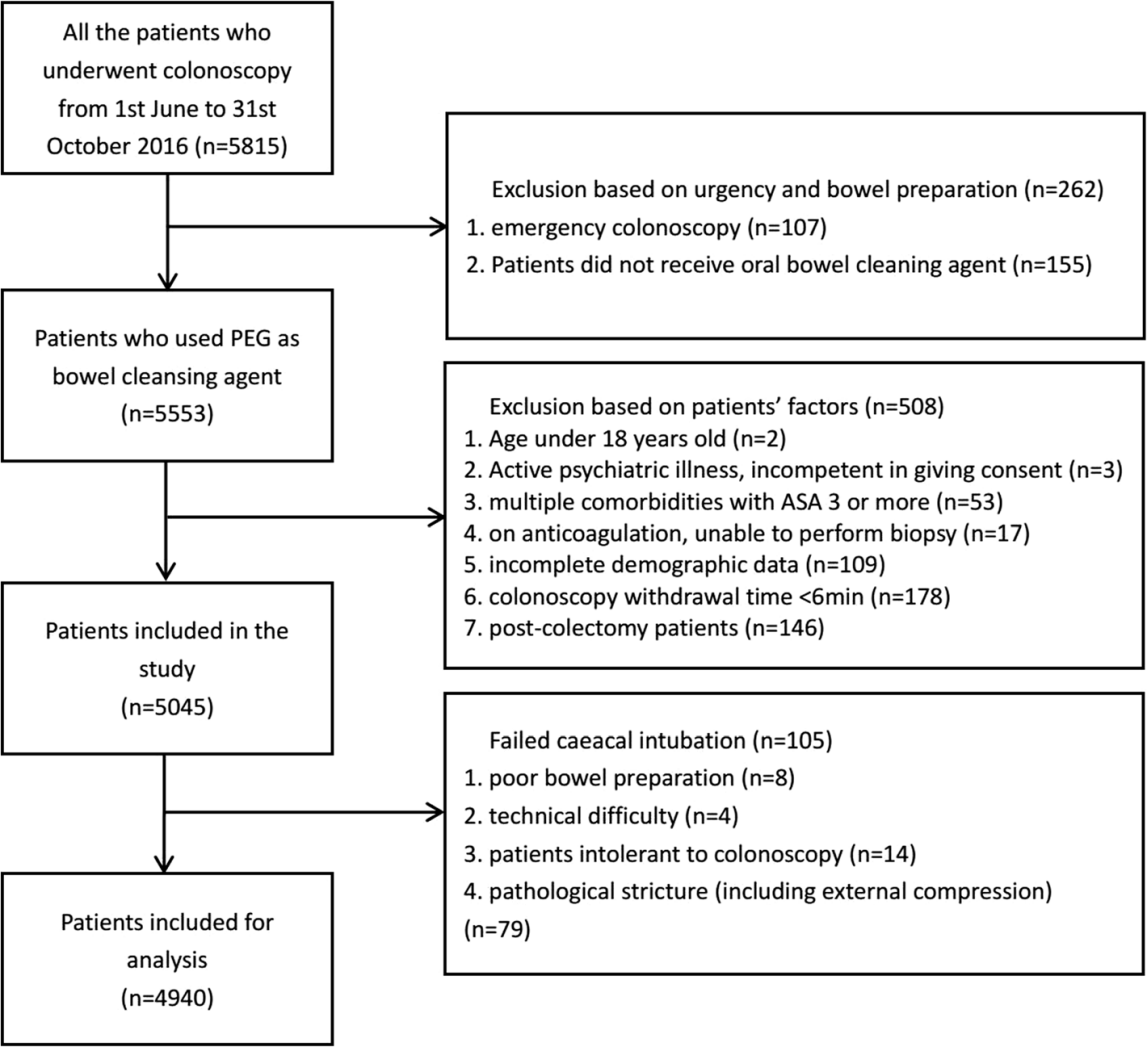
Study Flow

There were 5798 patients who underwent colonoscopy during the study period. 875 patients were excluded based on the exclusion criteria. 4940 patients’ colonoscopies (amount to 14,820 colon segments) were included in the final analysis (Fig. 1).

The mean age for the study population were 61.4 ± 10.3 years-old. 48.2% (2380/4940) were male and 51.8% (2560/4940) were female. 84.7% (4186/4940) of the indications of colonoscopy were for screening, surveillance and diagnostic purposes and the remnants were done for therapeutic purposes including endoscopic resection of lesions and polypectomy. Lesions detected in 2115 colonoscopies (42.8%). 1231 were adenomatous polyps (24.9%) and 476 were advanced adenomatous polyps (9.6%), (Table 1).

**Table 1:** Baseline Characteristics of the Study Participants

Patients,n	4940
Age,years,mean ± SD	61.4 ± 10.3
Male(%)	2380 (48.2)
Indication(%)	
Screening, Surveillance, Diagnostic	4186 (84.7)
For polypectomy	314 (6.4)
For ESD	440 (8.9)
Time of day colonoscopy performed(%)	
Morning	1236 (25.0)
Afternoon	3704 (75.0)
Participating Endoscopists(%)	
Senior	1575 (31.9)
Intermediate	2063 (41.8)
Junior	1302 (26.3)
Total BBPS score(%)	
0–3(inadequate bowel preparation)	101 (2.1)
4–5(poor bowel preparation)	885 (17.9)
6(fair bowel preparation)	1199 (24.3)
7–8(good bowel preparation)	2381 (48.2)
9(excellent or perfect bowel preparation)	374 (7.6)
Air bubble score(%)	
0(no air bubble)	1693 (34.3)
1(minimal bubbles)	1824 (36.9)
2(moderate amount of air bubbles)	984 (19.9)
3(large amount of air bubbles)	439 (8.9)
Colonoscopic findings(%)	2115 (42.8)
Adenoma	1231 (24.9)
Advanced adenoma	476 (9.6)
Cancer	104 (2.1)
Inflammatory bowel disease.	100 (2.0)
Diverticula	93 (1.9)
Others	111 (2.2)

The colonoscopy examination was performed by 42 endoscopists. 31.9% (1575/4940), 41.8% (2063/4940) and 26.3% (1302/4940) of the colonoscopies were done by senior, intermediate, and junior endoscopists, respectively. ADR and AADR were up to 28.4% (301/1059) and 6.4% (68/1059) respectively in senior endoscopists group while they were 18.9% (245/1295) and 2.8% (36/1295) in junior group. The inter-observer difference in using BBPS and BBS scoring among different categories of endoscopists were negligible (*P* = 0.149). However, there were statistical difference in ADR (*P* < 0.001) and AADR (*P* < 0.001) between different categories of endoscopists based on seniority.

Primary Outcome: The effect of BBPS in segments of bowel towards ADR and AADR.

BBPS classified the bowel cleanliness based on the score of 0 to 3 from worst to excellent bowel preparation for colon segments. 30.9% (4578/14820) scored 3, 57.5% (8522/14820) scored 2, 11.2% (1657/14820) scored 1 and 0.4% (63/14820) scored 0 for each score the ADR were 10.8% (493/4578), 7.7% (655/8522), 4.9% (81/1657) and 3.2% (2/63), respectively; whereas the AADR were 4.5% (207/4578), 2.8% (239/8522), 1.8% (29/1657) and 1.6% (1/63) (*P* < 0.05), (Table 2).

**Table 2:** Detection Rates and Differences in Detection Rates for Different Levels of Preparation Quality Based on BBPS Segment Scores. Effect of Colonic Segments’ BBPS on ADR

A. Effect of Colonic Segments’ BBPS on ADR					
	Segments’ score	ADR	Adjusted ADR	Comparison with BPPS	Adjusted ADR difference (95%CI)
Right-sided colon	BBPS = 0	2/56 (3.6%)	3.6%	BBPS2vs3	-0.4% (- 0.9to 0.1%)
	BBPS = 1	60/1214 (4.9%)	4.9%	BBPS1vs3	-2.3% (- 2.8%to-1.8%)^*^
	BBPS = 2	221/3243 (6.8%)	6.9%	BBPS1vs2	-1.9% (- 2.1%to-1.7%)^*^
	BBPS = 3	31/427 (7.3%)	7.3%	BBPS0vs2	-3.3% (- 3.8%to-2.7%)^*^
				BBPS0vs1	-1.4% (- 2.2%to-0.5%)^*^
Transverse colon	BBPS = 0	0/6 (0.0)	0.0%	BBPS2vs3	-0.9% (- 1.3%to-0.6%)^*^
	BBPS = 1	15/356 (4.2%)	4.2%	BBPS1vs3	-4.9% (- 5.3%to-4.5%)^*^
	BBPS = 2	279/3407 (8.2%)	8.2%	BBPS1vs2	-4.0% (- 4.3%to- 3.7%)^*^
	BBPS = 3	107/1171 (9.1%)	9.1%	BBPS0vs2	-8.2% (- 8.4%to-8.0%)^*^
				BBPS0vs1	-4.2% (- 4.5%to-4.0%)^*^
Left-sided colon (sigmoid-rectum)	BBPS = 0	0/1 (0.0%)	0.0%	BBPS2vs3	-3.7% (-4.0%to-3.3%)^*^
	BBPS = 1	6/87 (6.9%)	6.9%	BBPS1vs3	-5.0% (-5.7%to-4.4%)^*^
	BBPS = 2	155/1872 (8.3%)	8.3%	BBPS1vs2	-1.4% (-2.1%to-0.7%)^*^
	BBPS = 3	355/2980 (11.9%)	12.0%		
All the colonic segments	BBPS = 0	2/63 (3.2%)	3.2%	BBPS2vs3	-3.0% (- 3.3%to-2.9%)^*^
	BBPS = 1	81/1657 (4.9%)	4.9%	BBPS1vs3	-5.9% (-6.1%to-5.7%)^*^
	BBPS = 2	655/8522 (7.7%)	7.7%	BBPS1vs2	-2.8% (- 3.0%to-2.6%)^*^
	BBPS = 3	49/4578 (10.8%)	10.8%	BBPS0vs2	-4.5% (- 5.0%to-4.0%)^*^
				BBPS0vs1	-1.7% (- 2.2%to-1.2%)^*^
B. Effect of Colonic Segments’ BBPS on AADR					
	Segments’ score	AADR	Adjusted AADR	Comparison with BPPS	Adjusted AADR difference (95% CI)
Right-sided colon	BBPS = 0	1/56 (1.8%)	1.8%	BBPS2vs3	-0.2% (- 0.7to 0.3%)
	BBPS = 1	21/1214 (1.7%)	1.7%	BBPS1vs3	-1.8% (- 2.3%to-1.3%)^*^
	BBPS = 2	107/3243 (3.3%)	3.3%	BBPS1vs2	-1.6% (- 1.8%to-1.3%)^*^
	BBPS = 3	15/427 (3.5%)	3.5%	BBPS0vs2	-1.5% (- 2.0%to-1.0%)^*^
				BBPS0vs1	0.1% (- 0.6to 0.7%)
Transverse colon	BBPS = 0	0/6 (0.0%)	0.0%	BBPS2vs3	-0.9% (- 1.1%to-0.7%)^*^
	BBPS = 1	6/356 (1.7%)	1.7%	BBPS1vs3	-1.5% (- 1.8%to-1.2%)^*^
	BBPS = 2	77/3407 (2.3%)	2.3%	BBPS1vs2	-0.6% (- 0.8%to-0.3%)^*^
	BBPS = 3	37/1171 (3.2%)	3.2%	BBPS0vs2	-2.3% (- 2.4%to-2.2%)^*^
				BBPS0vs1	-1.7% (- 3.3%to-0.0%)^*^
Left-sided colon (sigmoid-rectum)	BBPS = 0	0/1 (0.0%)	0.0%	BBPS2vs3	-2.3% (- 2.6%to-1.9%)^*^
	BBPS = 1	2/87 (2.3%)	2.3%	BBPS1vs3	-2.9% (- 3.7%to-2.1%)^*^
	BBPS = 2	55/1872 (2.9%)	2.9%	BBPS1vs2	-0.6% (- 0.9%to-0.3%)^*^
	BBPS = 3	155/2980 (5.2%)	5.2%		
All the colonic segments	BBPS = 0	1/63 (1.6%)	1.6%	BBPS2vs3	-1.7% (- 1.9%to-1.5%)^*^
	BBPS = 1	29/1657 (1.8%)	1.8%	BBPS1vs3	-2.8% (- 3.0%to-2.6%)^*^
	BBPS = 2	239/8522 (2.8%)	2.8%	BBPS1vs2	-1.1% (- 1.2%to-0.9%)^*^
	BBPS = 3	207/4578 (4.5%)	4.5%	BBPS0vs2	-1.2% (- 1.6%to-0.8%)^*^
				BBPS0vs1	-0.2% (- 0.7to 0.4%)

^*^Statistically significant, *P* < 0.05

In general, when comparing BBPS of individual segment higher score of BBPS shows significantly higher ADR and ADDR (*P* < 0.05). However, there were several control groups that did not show statistical significant difference in term of ADR and AADR. At the right-sided colon, there was no difference in ADR between BBPS 2 and 3, AADR between BBPS 2 and 3, and AADR between BBPS 0 and 1.

Secondary Outcome: The effect of overall BBPS and BBS towards ADR and AADR.

All the colonoscopy performed were given an overall BBPS from 0 to 9 (worst to excellent bowel preparation) for total colon by summing up the bowel segments’ scores. The proportion from colonoscopy which scored from ≤3 and 4–5, 6, 7, 8 and 9 were 2.1% (101/4940), 17.9% (885/4940), 24.3% (1199/4940), 32.8% (1620/4940), 15.4% (761/4940), and 7.6% (374/4940) respectively. Most of the colonoscopies’ BBPS scored 6 and above 80% (3954/4940) and majority scored 7. The higher the BBPS score was, the higher ADR and AADR we got.

**Fig. 2:**
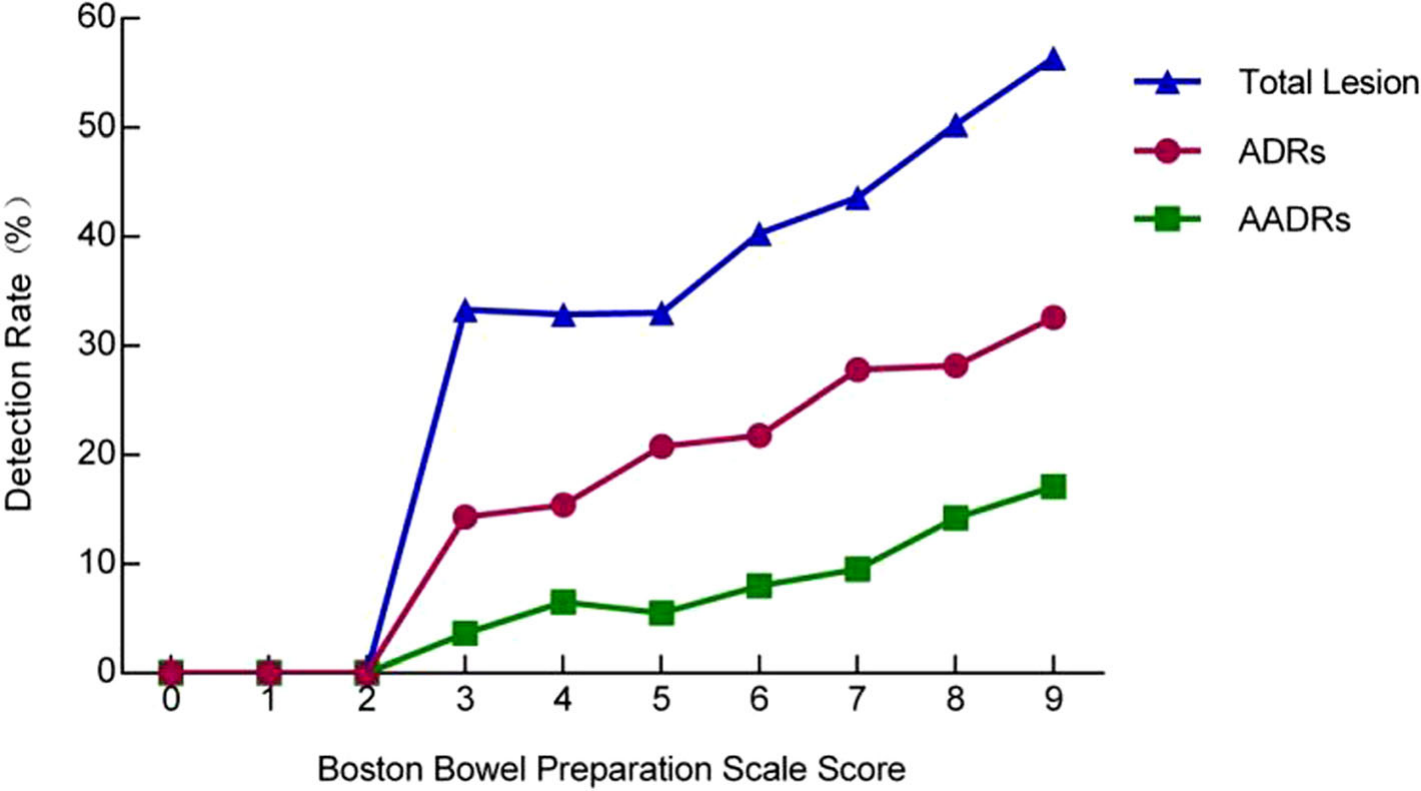
Detection Rates For Total Lesion, Adenoma and Advanced Adenoma for Different Levels Based on BBPS. Note: The influence of BBPS total score (0–9) on detection rate of adenoma, high-risk adenoma and total lesions was statistically significant. (*P* < 0.001)

The colonoscopies’ BBPS score ≤ 3 and ascending from 4 to 9 displayed rising ADR of 11.9% (12/101), 15.4% (38/246), 20.8% (133/639), 21.8% (261/1199), 27.8% (450/1620), 28.2% (215/761), and 32.6% (122/374), respectively, and the difference was statistically significant (Chi-square value 59.217, *P* < 0.001). Similarly, overall increasing AADR trend was observed from score of ≤3 and ascending from 4 to 9 were 3.0% (3/101), 6.5% (16/246), 5.5% (35/639), 8.0% (96/1199), 9.5% (154/1620), 14.2% (108/761) and 17.1% (64/374), respectively, and the difference was statistically significant (Chi-square value 66.661, *P* < 0.001) (Fig. 2).

**Fig. 3:**
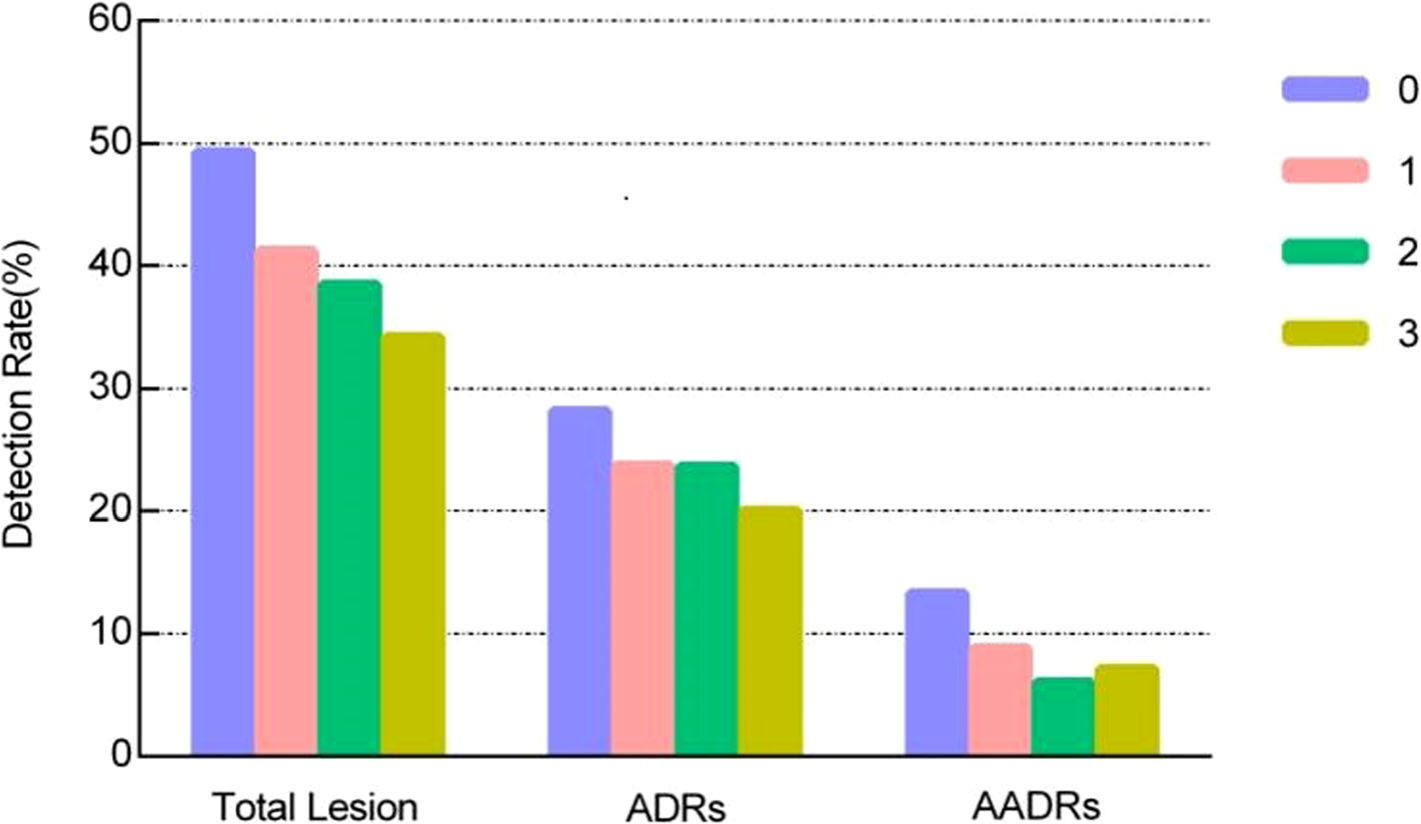
Detection Rates for Total Lesion, Adenoma and Advanced Adenoma for Different Levels Based on BBS

Bowel bubble score were given for all the colonoscopies from 0 to 3 (no bubble in sight to≥50% of view obscured by bubble). The proportion of BBS 0, 1, 2, and 3 were 34.3% (1693/4940), 36.9% (1824/4940), 19.9% (984/4940) and 8.9% (439/4940). The lower the BBS score was (the less the luminal bubbles), the higher the ADR and AADR we got. The ADR of colonoscopy with BBS of 0, 1, 2 and 3 were 28.2% (478/1693), 23.7% (433/1824), 23.6% (232/984) and 20.0% (88/439) respectively. Similarly, the AADR of colonoscopy with BBS 0, 1, 2, and 3 were 13.3% (226/1693), 8.8% (160/1824), 6.0% (59/984) and 7.1% (31/439) respectively (Fig. 3).

## Discussion

Optimizing bowel cleansing for colonoscopy is an important facet of successful colonoscopy surveillance programme. Guidelines recommend endoscopists to repeat colonoscopy when overall bowel preparation is inadequate because of high risk of missing polpys. The evidence to support this practice is scarce. Instead of focusing on overall bowel preparation, we assessed the importance of bowel cleanliness segmentally and correlate them with ADR and AADR of the same segments. In addition, we included overall colonoscopy bubble scoring in our study and correlate it with ADR and AADR in the similar manner. Our study found that the higher the segmental BBPS was (i.e. the better bowel preparation), the higher the ADR and AADR showed. Similarly, the lower the BBS score was (i.e. less bowel bubble), the higher the ADR and AADR were. Therefore we concluded that it would be beneficial to repeat colonoscopy to avoid missing adenomatous lesions when any segment of the BBPS is poor or contains large amount of bubbles, even overall bowel preparation is good or fair.

We used BBPS as standard assessment tool for bowel preparation. Comparing to Aronchick Bowel Preparation Scale and Ottawa Bowel Preparation Quality Scale, BBPS focuses on segmental assessment of bowel preparation. Moreover, it emphasizes on the assessment of bowel preparation after the attempt by endoscopists to cleanse bowel under endoscopy. Our study showed that when there is good bowel preparation with higher overall BBPS score, the ADR and AADR are higher. Jain D et al [22] indicated that AADR for bowel preparation with BBPS 7–9 was 16.7%, for BBPS = 4–6 was 14.8% and for BBPS = 0–3 was only 3.8%. There was statistically significant difference for the 3 groups (*P* < 0.05). In 2009, Lai EJ et al [19] demonstrated the correlation of overall BBPS and polyp detection rate. The study found that when the total score of BBPS was≥5, the ADR was 40% and if the BBPS was< 5, the ADR was only 24% (*P* < 0.02). Subsequently in 2010, Calderwood AH et al [23] introduced the BBPS for bowel segments’ preparation assessment. This study verified the use of BBPS as an objective and reproducible tool in this field. At the same time, they concluded that higher BBPS segment scores (2 and 3 versus 0 and 1) were associated with improved polyp detection rate at the left colon (OR 2.58, 95%CI 1.34–4.98) and right colon (OR 1.60, 95%CI 1.01–2.55). These results supported our primary outcomes.

To clarify the recommendation on follow-up for surveillance colonoscopy based on overall and segmental assessment of bowel preparation with BBPS score, Calderwood AH et al [24] conducted another study which showed that if the overall BPPS≥6 and individual bowel segment BBPS ≥2, the bowel preparation were considered adequate and the follow-up colonoscopy should be planned according to the standard colonoscopy surveillance guideline. Meanwhile, if total scores ≤2, it was regarded as inadequate and a repeated colonoscopy was highly recommended within 1 year. For colonoscopy with overall BBPS 3–5 (fair bowel preparation), Clark BT et al [25] had used the segmental bowel assessment to further decide the follow-up time for colonoscopy. The study particularly focused on the association of poor segmental bowel preparation with missed diagnosis of polyps. They concluded that patients with any colon segment score of < 2 should take an early repeat colonoscopy whereas those with all segment score ≥ 2 can repeat the surveillance colonoscopy based on standard guideline. In our study, when segmental bowel preparation (low BBPS score) were poor, ADR and AADR in the corresponding segment were low, and vice versa. Hence, we suggest repeating colonoscopy to reduce the risk of any polyp missing when the segments of bowel are poorly prepared.

Most of studies on bowel bubbles assessment were done on patients who underwent capsule endoscopy, with a standard bowel bubble score [20]. BBS has good reliability and reproducibility. Based on a previous study, 32–57% of colon had significant bowel bubbles which potentially obscure the view of endoscopy in China [18]. Compared to faecal materials and residues, bubbles are difficult to be cleared with conventional endoscopic washing and aspirating. We attempted to extrapolate the use of such score in large bowel examination and managed to demonstrate that it does affect ADR and AADR. We utilized BBS in addition to BBPS for bowel cleanliness assessment. In our study, 65.7% (3247/4940) of patients had more or less bubbles that affect the quality of endoscopic examination. We found that the higher the BBS score was (more bubbles), the lower the ADR and AADR were. So it is imperative to repeat colonoscopy if the BBS score is high.

There were several limitations in our study. Firstly, the study did not restrict the selection of patients to those whom were colonoscopy naïve. These included the patient who had underwent previous colonoscopy and planned for therapeutic polypectomy or endoscopic resection. This would affect the ADR and AADR for the repeat cases obviously, as the lesion is known before the examination. Nevertheless, the statistical calculation in the study had focused on the association between bowel preparation and adenomatous lesion detection rather than discovery of these lesions per se. Furthermore, there is no evidence to show that bowel cleanliness is affected by the number of colonoscopy that was performed in the same patient. We believe that the inclusion of the repeat cases in the study had minimal effect on the correlation.

Secondly, the inadequacy in number of study population in certain categories has resulted in failure in demonstrating the difference on the ADR and AADR in a few groups. If the number of patients had been greater, it might have been able to show a difference, which large sample studies are needed for further testified.

Thirdly, the assessment of BBS score can be improved by performing segmentally and by using simethicone [14, 15]. We have observed that colonic bubbles mainly occurred in right colon and transverse colon and it is well-established that polyp missing rate, especially sessile lesion which carries high risk of malignant transformation, is unacceptably high in this segment of colon. Meanwhile, it is ideal to use simethicone as suggested by several guidelines to reduce luminal bubbles in order to improve the endoscopic view. However, simethicone is not a routine in China, particularly the awareness of its importance is not widespread. As a result, the data that we obtained in the study reflected the real-world practice locally. Thus we believe that a follow up study assessing the bubble scoring segmentally, with further intervention to reduce luminal bubbles should be done to demonstrate the significant correlation of reducing luminal bubbles with improvement of ADR/AADR.

Finally, this is a single-centered and cross-sectional study. Despite of the statistical adjustment, there was inherited bias due to the existence of many other confounding factors. This can be improved by conducting a multi-centered prospective trial.

## Conclusions

Our study illustrates that the cleaner (the higher BBPS score) the individual segment of bowel was, and the less amount of overall luminal bowel bubbles existed (the lower the BBS score), the higher ADR and AADR were. We concluded that a repeat colonoscopy should be planned if any segment of the bowel is poorly prepared, or if there is more bubbles, even if the overall assessment score of the bowel preparation is good or fair.

## Data Availability

All data generated or analysed during this study are included in this published article. The datasets generated and analysed during the current study are available from the corresponding author by email zhaihuihong@263.net on reasonable request.

## Abbreviations

AADR: Advanced Adenoma Detection Rate
ACG: American College of Gastroenterology
ADR: Adenoma Detection Rate
AGA: American Gastroenterological Association
ASA: American Society of Anaesthesiology
ASGE: American Society Gastrointestinal Endoscopy
BBPS: Boston Bowel Preparation Scale
BBS: Bowel Bubble Scale
EMR: Endoscopic Mucosal Resection
ESD: Endoscopic Submucosal Dissection
NBI: Narrow Band Imaging
PEG: Polyethylene Glycol-electrolyte Solution
